# High resolution crystal structures of the receptor-binding domain of *Clostridium botulinum* neurotoxin serotypes A and FA

**DOI:** 10.7717/peerj.4552

**Published:** 2018-03-21

**Authors:** Jonathan R. Davies, Gavin S. Hackett, Sai Man Liu, K. Ravi Acharya

**Affiliations:** 1 Department of Biology and Biochemistry, University of Bath, Bath, United Kingdom; 2 Ipsen Bioinnovation Limited, Abingdon, United Kingdom

**Keywords:** SV2, Crystal structure, Botulinum neurotoxin, Targeted secretion inhibitor, FA hybrid, Receptor binding domain

## Abstract

The binding specificity of botulinum neurotoxins (BoNTs) is primarily a consequence of their ability to bind to multiple receptors at the same time. BoNTs consist of three distinct domains, a metalloprotease light chain (LC), a translocation domain (H_N_) and a receptor-binding domain (H_C_). Here we report the crystal structure of H_C_/FA, complementing an existing structure through the modelling of a previously unresolved loop which is important for receptor-binding. Our H_C_/FA structure also contains a previously unidentified disulphide bond, which we have also observed in one of two crystal forms of H_C_/A1. This may have implications for receptor-binding and future recombinant toxin production.

## Introduction

Botulinum neurotoxin (BoNT) is the active agent that causes the deadly condition botulism. It is expressed as a single polypeptide chain of approximately 150 kDa and is cleaved post-translationally to yield an active di-chain molecule held together by a single disulphide bond. The smaller 50 kDa light chain (LC) possesses a single zinc-endopeptidase domain whereas the larger 100 kDa heavy chain is comprised of two domains—a target cell receptor binding (H_C_) domain and a translocation (H_N_) domain (Schiavo et al., 1992; Montecucco, 1986). BoNTs are the most poisonous biological substances known to man and their remarkable toxicity is a result of their highly specific and modular mechanism of action. The toxins target neuronal cell membranes through the formation of a dual receptor binding complex (Montecucco, 1986) which allows for internalisation by endocytosis (Colasante et al., 2013). The H_N_ domain then translocates the LC into the cytoplasm where it cleaves a specific SNARE (soluble N-ethylmaleimide-sensitive factor attachment protein receptor) protein which consequently inhibits vesicle release. Many immunologically distinct BoNTs have been discovered over the years—serotypes /A through to /G; although, only serotypes /A, /B, /E and /F have been reported to cause botulism in humans (Coffield et al., 1997). Each serotype can be further subdivided into different subtypes based on their amino acid sequence (Rossetto, Pirazzini & Montecucco, 2014).

The BoNT H_C_ domain is responsible for targeting the toxin to a specific cell type and the specific receptors involved have been identified for most serotypes. For example, serotype /A binds to a glycosylated luminal domain of the synaptic vesicle 2 (SV2) protein, preferentially to the C isoform (SV2C) (Dong et al., 2006; Mahrhold et al., 2006), as well as a ganglioside, namely GT1b (Rummel et al., 2004; Yowler & Schengrund, 2004; Stenmark et al., 2008). X-ray crystallography has revealed protein-backbone hydrogen-bond interactions between β-strands of the BoNT/A1 H_C_ and the fourth luminal domain of SV2C (SV2C-LD4), as well as electrostatic interactions between charged surfaces (Benoit et al., 2014). This is supplemented by additional interactions with the N-linked glycans present on the native SV2 molecule—this network of glycan interactions is key for BoNT potency (Yao et al., 2016).

BoNTs have been exploited for therapeutic use in many neurological indications such as dystonia and overactive bladder (Dressler, 2012). More recently, BoNTs have been re-engineered to target different cell types and treat specific clinical indications (Masuyer et al., 2014; Masuyer et al., 2015)—these are known as targeted secretion inhibitors (TSIs). A detailed understanding of the structural aspects of the different BoNT subtypes will prove useful in identifying regions of variability that may help uncover conserved mechanisms of binding, which in turn will guide efforts in developing novel BoNT therapeutics. Therefore, high-resolution, three-dimensional structural analysis of naturally occurring BoNTs, their mosaics and subtypes, would be of great value. Of particular interest are the structural differences arising from sequence differences between subtypes.

Recently, a new bivalent strain of *Clostridium botulinum,* IBCA10-7060, was reported to produce BoNT/B2 and a previously unknown BoNT serotype—“BoNT/H” (Barash & Arnon, 2014). This novel toxin is a mosaic molecule and is now more commonly referred to as BoNT/FA (as well as BoNT/HA) due to a LC similar to that of BoNT/F5, a H_N_ domain similar to that of BoNT/F1, and a H_C_ domain similar to that of BoNT/A1 (Maslanka et al., 2015; Gonzalez-Escalona et al., 2014; Kalb et al., 2015). The crystal structure of the BoNT/FA binding domain was recently reported (Yao et al., 2017). Here, we present a new crystal structure of H_C_/FA at 1.95 Å resolution which reveals further structural information that is unresolved in the reported structure. Specifically, our structure reveals a loop previously unmodeled due to lack of density, which is of high importance, and we also observe a disulphide bond which was not present within the previous structure. To this end we have produced two crystal forms of H_C_/A1 (determined to 1.45 Å and 1.7 Å respectively) differing by the presence of this disulphide bond.

## Materials and Methods

### Cloning and constructs

The genes encoding the binding domain of BoNT/A1 (H_C_/A1) and BoNT/FA (H_C_/FA) were provided by Ipsen Bioinnovation Ltd. Each was cloned into the pJ401 expression vector (DNA 2.0, Menlo Park, CA, USA) with an N-terminal 6× histidine tag using standard molecular biology techniques and confirmed by sequencing (Eurofins Genomics, Germany).

### Protein expression

His_6_-H_C_/A1 and His_6_-H_C_/FA were expressed in *E. coli* strain BL21(DE3) (Novagen, Madison, WI, USA) using the following protocol. A glycerol stock was used to inoculate 100 mL TB medium containing 50 µg/mL kanamycin, and grown at 37 °C for 16 h. From this, 10 mL of culture was used to inoculate 1 L TB medium containing 50 µg/mL kanamycin and grown at 37 °C to an OD_600_ of 0.6. The temperature was reduced to 16 °C and the cells grown to an OD_600_ of 1.0 at which point 0.5 mM isopropyl-d-1-thiogalactopyranoside (IPTG) was added to induce expression. Cells were grown for an additional 16 h at 16 °C and then harvested by centrifugation at 4,000× g for 30 min.

### Protein purification

Expression cell pastes were resuspended in 0.5 M NaCl, 50 mM Tris–HCl pH 7.4, 20 mM imidazole and lysed using a Constant Systems cell disruptor at 20 kPSI. Cell debris were removed by centrifugation at 80,000× g for 30 min and the supernatant was filtered through a 0.22 µm membrane syringe filter. The clarified lysate was loaded onto a 5 mL HisTrap column (GE Healthcare, Little Chalfont, UK), washed, and the target protein eluted with 0.5 M imidazole. His_6_-H_C_/A1 and His_6_-H_C_/FA were further purified by SEC using a Superdex 200 16/60 column (GE Healthcare, Little Chalfont, UK) and 0.5 M NaCl, 50 mM Tris-HCl pH 7.4. Purified samples were concentrated to 10 mg/mL using a 10 kDa MWCO centrifugal filter (Millipore, Billerica, MA, USA).

### Crystallography

Crystals of His_6_-H_C_/FA and His_6_-H_C_/A1 were grown at 16 °C using a 1:1 ratio of protein solution (10 mg/mL) to well solution using the sitting-drop vapour-diffusion method—4 M sodium formate, 0.1 M sodium acetate pH 5.5 for the former, and 0.1 M MIB pH 4.0, 25% w/v PEG 1500 for the latter. Crystals were soaked in cryoprotectant (equal volume of reservoir solution and 50% glycerol) before vitrification in liquid nitrogen. Complete datasets were collected on beamline I03 and I04, respectively, at the Diamond Light Source (Didcot, UK). Diffraction images were processed using DIALS (Gildea et al., 2014) and scaled using AIMLESS (Evans & Murshudov, 2013) from the CCP4 suite (Winn et al., 2011). Data collection statistics are summarised in Table 1. A combination of R_pim_ and CC_1/2_ value were used to determine the resolution cut-off of 1.95 Å and 1.45 Å, respectively. Phase information was determined by molecular replacement using PHASER (McCoy et al., 2007) and a previous structure of H_C_/A1 (PDB: 2VUA; Stenmark et al., 2008) as the initial search model. Multiple rounds of structure refinement were performed by manual correction in COOT (Emsley et al., 2010) followed by restrained refinement with REFMAC5 (Murshudov et al., 2011). Final validation was performed with MolProbity (Chen et al., 2010). Secondary structures were annotated using Stride (Frishman & Argos, 1995) and figures were prepared using PyMol (Schrödinger, LLC, New York, NY, USA) and CCP4mg (McNicholas et al., 2011).

**Table 1:** Crystallographic data collection and refinement statistics.

	H_C_/FA	H_C_/A1 (crystal form 1)	H_C_/A1 (crystal form 2)
Space Group	P422	P2_1_2_1_2_1_	P2_1_
Cell dimensions			
*a, b, c* (Å)	118.0, 118.0, 173.8	39.8, 107.3, 107.6	61.4, 53.9, 62.7
*α*, *β*, *γ* (°C)	90.0, 90.0, 90.0	90.0, 90.0, 90.0	90.0, 106.1, 90.0
Resolution (Å)	24.40–1.95 (1.98–1.95)^a^	24.10–1.45 (1.48–1.45)^a^	60.24–1.80 (1.84–1.80)^a^
R_merge_ (%)	20.1 (168.6)^a^	7.6 (69.2)^a^	14.2 (53.9)^a^
R_meas_ (%)	20.8 (176.0)^a^	9.7 (92.0)^a^	15.0 (58.8)^a^
R_pim_ (%)	5.6 (50.2)^a^	5.8 (60.1)^a^	4.6 (22.5)^a^
CC1/2	0.999 (0.832)^a^	0.997 (0.357)^a^	0.962 (0.954)^a^
<I/*σ*(I)>	12.6 (2.3)^a^	6.9 (1.3)^a^	14.4 (3.7)^a^
Completeness (%)	100.0 (100.0)^a^	95.3 (95.1)^a^	98.6 (89.1)^a^
Multiplicity	26.0 (23.7)^a^	3.3 (2.7)^a^	20.4 (11.7)^a^
R_work_ (%)	18.0	17.6	18.8
R_free_ (%)	20.9	22.1	22.3
No. of atoms			
Protein	6,907	3,511	3,263
Water	609	421	322
RMSD bond length (Å)	0.007	0.002	0.005
RMSD bond angle (°)	0.89	0.44	0.73
Wilson B factor (Å^2^)	24.6	15.9	13.4
Average B factors (Å^2^)			
Protein	28.2, 30.0	21.2	22.5
Solvent	33.8	34.1	28.0
Ramachandran plot			
Favoured (%)	96.7	96.3	96.4
Allowed (%)	3.4	3.5	3.6
Disallowed (%)	0.0	0.2	0.0
PDB code	5MK8	5MK6	5MK7

**Notes.**

a Values in parentheses are for the highest resolution shell.

## Results and Discussion

### Crystal structure of BoNT/FA H_C_ domain

**Figure 1:**
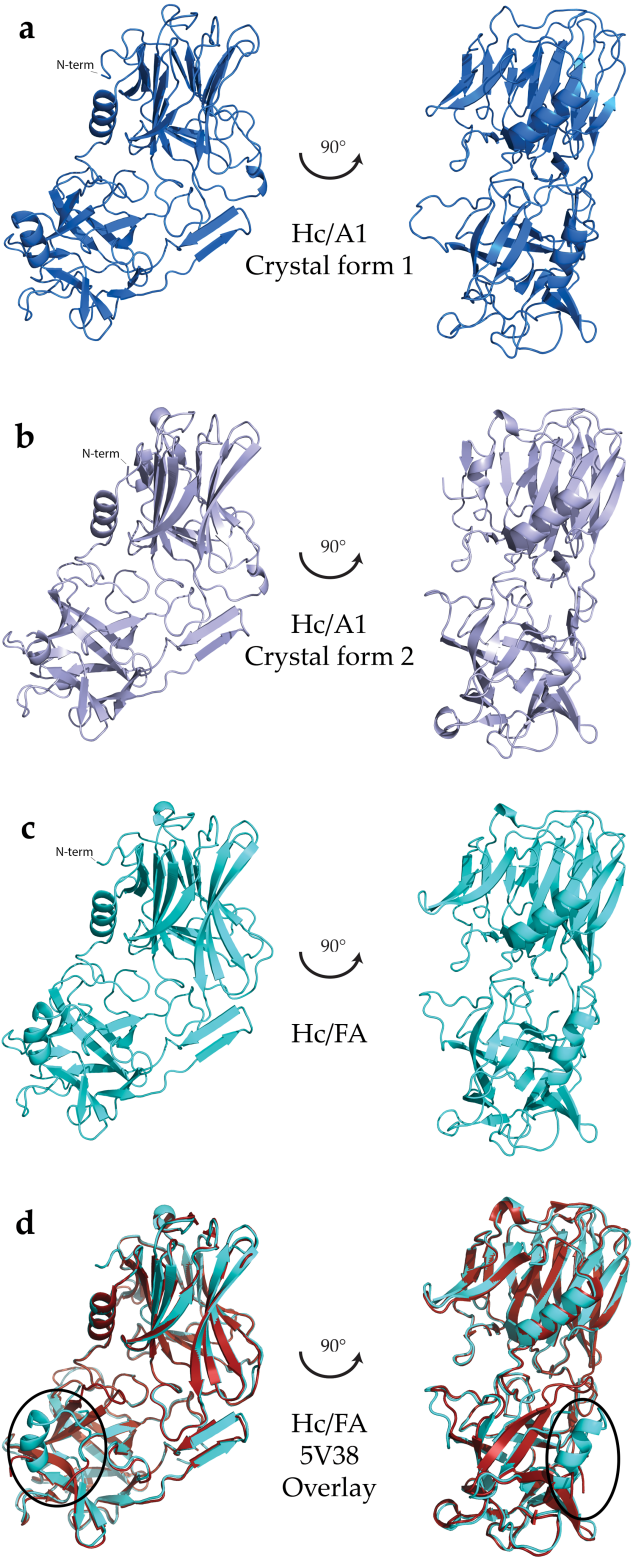
Crystal structures of HC domains. (A) HC/A1 domain (crystal form 1, PDB: 5MK6), (B) HC/A1 domain (crystal form 2, PDB: 5MK7), (C) HC/FA domain (PDB: 5MK8), and (D) overlay with a different crystal form of HC/FA (PDB:5V38; Yao et al., 2017). The position of loop R1261–R1268 indicated with an ellipse. All structures represented as a ribbon diagram, generated using PyMol (Schrödinger, LLC, New York, NY, USA).

**Figure 2:**
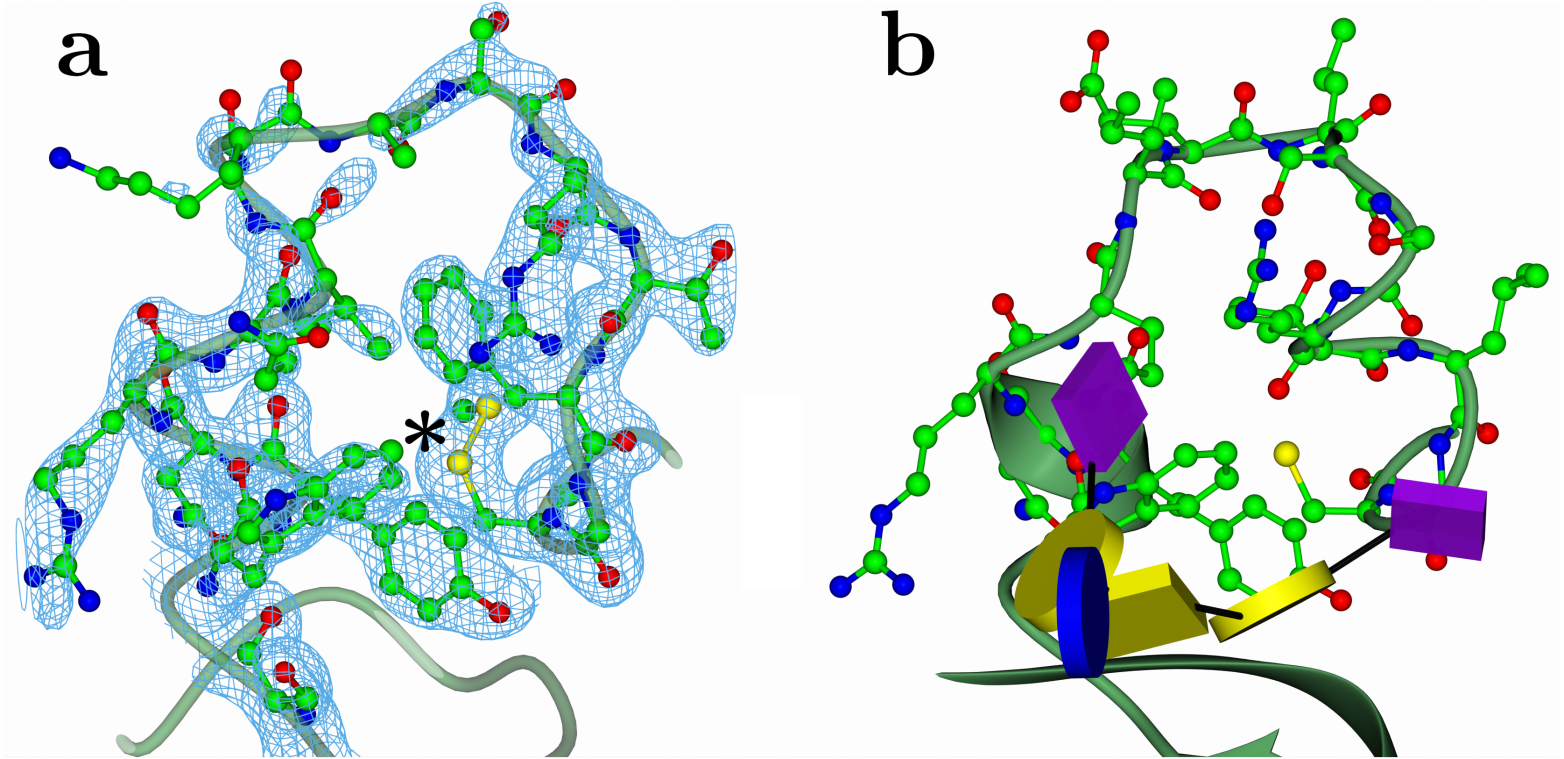
Ganglioside binding site. (A) Electron density from a composite omit map for HC/FA. The location of a disulphide bond is marked with an asterisk. (B) The equivalent loop from HC/A1 (2VU9) with GT1b shown in glycoblock representation. Map produced using Phenix package (Terwilliger et al., 2008).

We identified crystallisation conditions which yielded crystals of H_C_/FA in space group P422, with two molecules related by non-crystallographic symmetry in the asymmetric unit. This is different to a recently reported structure, PDB: 5V38 (Yao et al., 2017) and reveals an important loop that is involved in receptor binding (average temperature factor (B-factor) 74 Å^2^). A high-multiplicity dataset was collected containing 360 degrees of data over 3,600 images. No significant radiation damage was observed over the course of data collection and thus all data were used. The CC_1/2_ value for the outer shell was 0.832, indicating there was still very usable data at this resolution (Evans & Murshudov, 2013). The overall structure of H_C_/FA is shown in Fig. 1C and the crystallographic statistics are listed in Table 1. As with all reported structures of the BoNT receptor binding domain, present is a characteristic β-jellyroll fold at the N-terminal half and a predominantly β-trefoil fold at the C-terminal half of the protein (Figs. 1A–1D). Both molecules in the asymmetric unit overlay well with a root mean square deviation (RMSD) value of 0.35 Å between all atoms. The B-factors for each chain are low overall (24.74 Å^2^ and 26.84 Å^2^ respectively) with a corresponding overall Wilson B-factor of 24.55 Å^2^. As expected, our structure is highly similar to PDB: 5V38 with an RMSD value for combinations of chains between structures ranges from 0.54 Å to 0.36 Å (Fig. 1D). However, it further reveals the presence of a loop (R1261–R1268) that has been shown in other subtypes to be involved in ganglioside receptor binding (Fig. 2A). Crystal packing has enabled neighbouring chains to interact directly with this loop, provide sufficient stabilisation to produce good electron density. Yao et al. (2017) suggested that the lack of density was due to high flexibility, which is consistent with the high B-factors we observed in this region relative to the rest of the protein.

**Figure 3:**
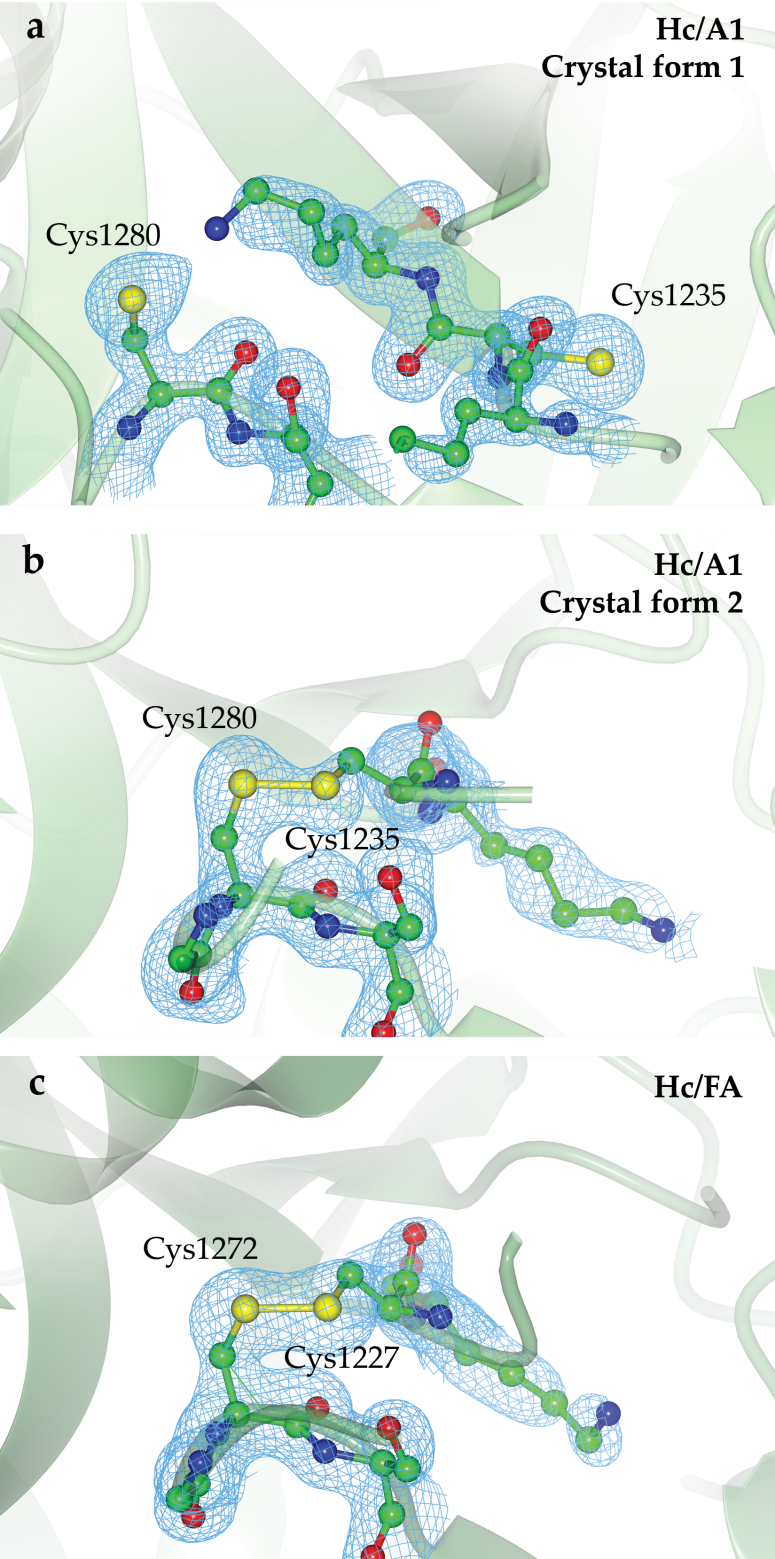
Location of Cys1235 and Cys1280 residues. (A) Cys1235 and Cys1280 are not covalently bound in the HC/A1 crystal form 1, (B) disulphide bond formation in crystal form 2 and (C) a similar disulphide bond is seen in the HC/FA crystal structure between equivalent cysteines. Composite OMIT maps were produced using Phenix (Terwilliger et al., 2008). And are shown for each model at 1 σ. Figure generated using CCP4mg (McNicholas et al., 2011).

The specific ganglioside receptor for BoNT/FA is not yet known; however, considering that GT1b binds with high affinity to BoNT/A1 (Fig. 2B) and that H_C_/FA and H_C_/A1 are structurally very similar, we propose that BoNT/FA possesses a similar binding specificity. The overall conformation of this region is also similar to that of BoNT/A1 in complex with a ganglioside receptor (Fig. 2). The detailed conformation of this loop is important for understanding receptor binding and our structure confirms that BoNT/FA could bind to gangliosides in a similar manner to BoNT/A. Proximate to the ganglioside binding region, we observe the presence of a disulphide bond between Cys1227 and Cys1272 (Fig. 3C) which is also not present in the structure 5V38. The equivalent bond has been observed previously in some, but not all crystal structures of BoNT H_C_ Domains; therefore, it is uncertain what role, if any, it may have towards BoNT function.

### Crystal structures of the BoNT/A1 binding domain

**Figure 4:**
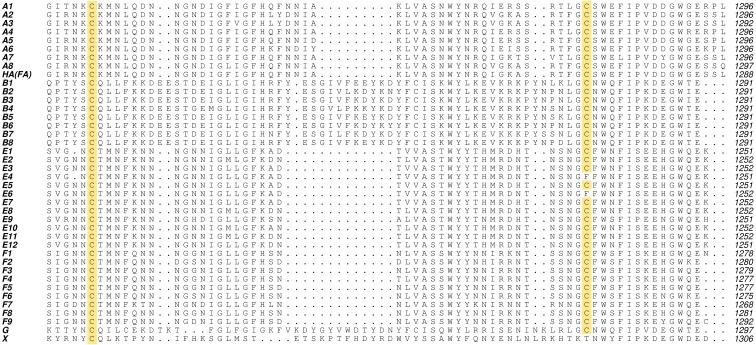
Amino acid sequence alignment of BoNT subtypes emphasising the strong conservation of two cysteine residues near the protein C-terminus. UniprotKB accession numbers—A1: A5HZZ9, A2: Q45894, A3: Q3LRX9, A4: Q3LRX8, A5: C1IPK2, A6: C9WWY7, A7: K4LN57, A8: A0A0A7PDB7, HA(FA): WP_047402807 *, B1: P10844, B2: A0A0B4W2B0, B3: A2I2S2, B4: A2I2S4, B5: A0A0E1L271, B6: H3K0G8, B7: H9CNK9, B8: M9VUL2, E1: Q00496, E2: A2I2S6, E3: A0A076L133, E4: P30995, E5: Q9K395, E6: A8Y878, E7: G8I2N7, E8: G8I2N8, E9: WP_017352936 *, E10: A0A076JVL9, E11: A0A076K0B0, E12: A0A0A7RCR1, F1: A7GBG3, F2: Q9ZAJ5, F3: D2KHR6, F4: D2KHQ8, F5: D2KHQ9, F6: D2KHS6, F7: D2KHS9, F8: WP_076177537 *, F9: A0A1P8YWK9, G: Q60393, X: WP_045538952 * (*indicates NCBI accession code where UniprotKB accession is not available).

We have identified a single crystallisation condition that produced two crystal forms of H_C_/A1—one possessed the equivalent disulphide bond whereas the other did not. Using 25% w/v PEG 1500 and 0.1 M MIB pH 4.0, H_C_/A1 crystallised into orthorhombic crystals with the space group P2_1_2_1_2_1_ (crystal form 1) that diffracted to a resolution of 1.45 Å. No disulphide bond was observed in this structure (Fig. 1A). Instead, Cys1235 rotated away from the junction due to a backbone rotation, bringing the neighbouring Lys1236 toward Cys1280 (Fig. 3A). This is consistent with previous H_C_/A1 structures either in complex with GT1b (PDB: 2VU9; Stenmark et al., 2008), SV2-LD (PDB: 4JRA, 5JLV; Benoit et al., 2014; Yao et al., 2016) or in the apo form (PDB: 2VUA; Stenmark et al., 2008). Using the same conditions, monoclinic crystals were obtained six months later with the space group P2_1_ (crystal form 2) that diffracted to 1.8 Å resolution. Inspection of this structure (Fig. 1B) revealed the presence of a disulphide bond between C1235 and C1280, the equivalent of which was also observed in our H_C_/FA crystal structure (Figs. 3A and 3B) and in a full-length BoNT/A1 crystal structure (PDB: 3BTA; Lacy & Stevens, 1999). Our findings suggest that the crystallisation condition is not the only determinant as to whether the bond is present or not. The conservation of these cysteine residues across the BoNT sub-serotypes suggests they are very important (Fig. 4); however, their precise function is unknown. Almost all BoNT subtypes that cause human botulism contain the two residues. They may be required for disulphide bond formation for structural stability or for efficient function of a nearby ganglioside-binding pocket. It must be noted that the expression system does not appear to select for reduced or non-reduced forms of these cysteine residues—each have been observed in different structures of BoNT/A purified from the native *Clostridium botulinum* (Garcia-Rodriguez et al., 2007; Stenmark et al., 2008; Fu et al., 2009; Gu et al., 2012; Przedpelski et al., 2013; Benoit et al., 2014; Yao et al., 2017; Davies et al., 2017).

## Conclusion

The high-resolution crystal structures of the binding domains (H_C_) from BoNT/FA and BoNT/A1 are reported here. The former complements a recently published structure (Yao et al., 2017) and resolves a loop which is highly important for receptor binding. For the latter, two structures were determined from two crystal forms obtained from the same crystallisation condition. These H_C_/A1 structures differed from one another by the presence or absence of a disulphide bond. This bond was also observed in our structure of H_C_/FA. Considering their location near the ganglioside-binding pocket, and conservation across BoNT subtypes, the redox status of these conserved cysteines may have implications in BoNT stability and manufacture. Botulinum neurotoxins are used therapeutically for many indications and their production is currently from the native host *Clostridium botulinum*. However, considering the safety implications and the advent of engineered BoNT derivatives, such as TSIs, production from a recombinant source would be highly desirable. The importance of these two cysteine residues is being further investigated.

##  Supplemental Information

10.7717/peerj.4552/supp-1 Supplemental Information 1 PDB coordinates files and validation reports

## Abbreviations

BoNT/X: botulinum neurotoxin serotype X
BoNT/FA: botulinum neurotoxin hybrid of /F and /A serotypes
H_C_: receptor binding domain of BoNT
LC: light chain of BoNT, catalytic domain
H_N_: translocation domain of BoNT
SV2C: synaptic vesicle 2 protein, isoform C
TSI: targeted secretion inhibitor

## Additional Information and Declarations

### Data Availability

The following information was supplied regarding data availability: Accession codes: The atomic coordinates and structure factors (codes 5MK6, 5MK7, 5MK8) have been deposited in the Protein Data Bank (http://www.pdb.org).
