# Supplementary material for: High resolution crystal structures of the receptor-binding domain of Clostridium botulinum neurotoxin serotypes A and FA
Source: PeerJ. 2018 Mar 21;6:e4552. doi: 10.7717/peerj.4552 (PMC5866713; doi:10.7717/peerj.4552)
Supplement: Supplemental Information 1 [file peerj-06-4552-s001.zip › D_1200002586_val-report-full-annotate_P1.pdf]

WORLDWIDE PDB PROTEIN DATA BANK

Full wwPDB X-ray Structure Validation Report ⓘ

CONFIDENTIAL VALIDATION REPORT

Dec 6, 2016 – 11:52 AM GMT

PDB ID	:	5MK8
Title	:	Crystal structure of the receptor-binding domain of the FA hybrid Clostridium botulinum neurotoxin
Deposited on	:	2016-12-02
Resolution	:	1.95 Å(reported)

This is a Full wwPDB X-ray Structure Validation Report.

This report is produced by the wwPDB biocuration pipeline after annotation of the structure.

We welcome your comments at *validation@mail.wwpdb.org*
A user guide is available at
http://wwpdb.org/validation/2016/XrayValidationReportHelp
with specific help available everywhere you see the ⓘ symbol.

The following versions of software and data (see references ⓘ) were used in the production of this report:

MolProbity	:	4.02b-467
Mogul	:	1.7.1 (RC1), CSD as537be (2016)
Xtriage (Phenix)	:	1.9-1692
EDS	:	rb-20028442
Percentile statistics	:	20151230.v01 (using entries in the PDB archive December 30th 2015)
Refmac	:	5.8.0135
CCP4	:	6.5.0
Ideal geometry (proteins)	:	Engh & Huber (2001)
Ideal geometry (DNA, RNA)	:	Parkinson et al. (1996)
Validation Pipeline (wwPDB-VP)	:	rb-20028442

1 Overall quality at a glance ⓘ

The following experimental techniques were used to determine the structure:
X-RAY DIFFRACTION

The reported resolution of this entry is 1.95 Å.

Percentile scores (ranging between 0-100) for global validation metrics of the entry are shown in the following graphic. The table shows the number of entries on which the scores are based.

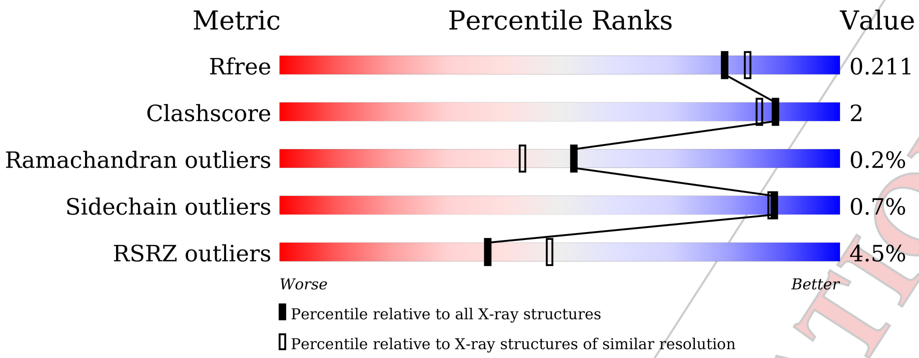

Metric	Whole archive (#Entries)	Similar resolution (#Entries, resolution range(Å))
R_{free}	91344	1833 (1.96-1.96)
Clashscore	102246	1953 (1.96-1.96)
Ramachandran outliers	100387	1936 (1.96-1.96)
Sidechain outliers	100360	1936 (1.96-1.96)
RSRZ outliers	91569	1835 (1.96-1.96)

The table below summarises the geometric issues observed across the polymeric chains and their fit to the electron density. The red, orange, yellow and green segments on the lower bar indicate the fraction of residues that contain outliers for >=3, 2, 1 and 0 types of geometric quality criteria. A grey segment represents the fraction of residues that are not modelled. The numeric value for each fraction is indicated below the corresponding segment, with a dot representing fractions <=5% The upper red bar (where present) indicates the fraction of residues that have poor fit to the electron density. The numeric value is given above the bar.

Mol	Chain	Length	Quality of chain
1	A	437	4% / 90% 5% •
1	B	437	5% / 89% 7% •

The following table lists non-polymeric compounds, carbohydrate monomers and non-standard residues in protein, DNA, RNA chains that are outliers for geometric or electron-density-fit criteria:

CONFIDENTIAL VALIDATION REPORT

Mol	Type	Chain	Res	Chirality	Geometry	Clashes	Electron density
2	FMT	A	1302	-	-	X	X
2	FMT	A	1303	-	-	-	X
2	FMT	A	1304	-	-	-	X
2	FMT	A	1305	-	-	-	X
2	FMT	B	1301	-	-	-	X
2	FMT	B	1302	-	-	-	X
2	FMT	B	1303	-	-	-	X
2	FMT	B	1305	-	-	-	X

CONFIDENTIAL VALIDATION REPORT

2 Entry composition (i)

There are 4 unique types of molecules in this entry. The entry contains 7553 atoms, of which 0 are hydrogens and 0 are deuteriums.

In the tables below, the ZeroOcc column contains the number of atoms modelled with zero occupancy, the AltConf column contains the number of residues with at least one atom in alternate conformation and the Trace column contains the number of residues modelled with at most 2 atoms.

- Molecule 1 is a protein called Botulinum neurotoxin FA binding domain.

Mol	Chain	Residues	Atoms					ZeroOcc	AltConf	Trace
1	A	421	Total 3458	C 2202	N 592	O 648	S 16	0	2	0
1	B	420	Total 3449	C 2194	N 591	O 648	S 16	0	2	0

- Molecule 2 is FORMIC ACID (three-letter code: FMT) (formula: CH_2O_2).

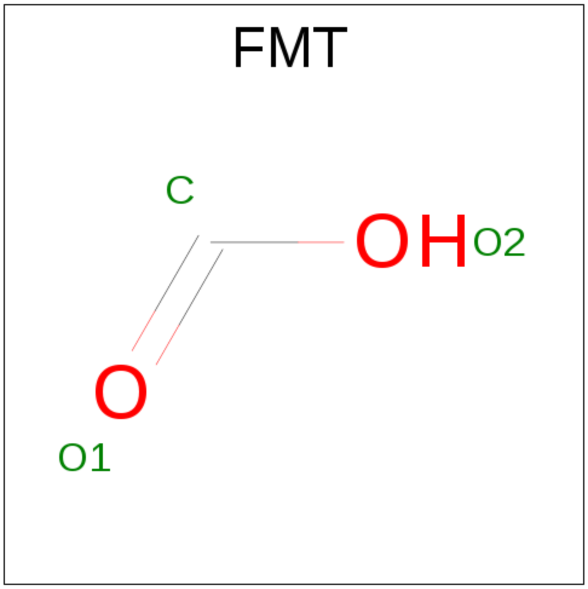

Mol	Chain	Residues	Atoms			ZeroOcc	AltConf
2	A	1	Total 3	C 1	O 2	0	0
2	A	1	Total 3	C 1	O 2	0	0
2	A	1	Total 3	C 1	O 2	0	0
2	A	1	Total 3	C 1	O 2	0	0

Continued on next page...

CONFIDENTIAL VALIDATION REPORT

Continued from previous page...

Mol	Chain	Residues	Atoms	ZeroOcc	AltConf
2	A	1	Total C O<br>3 1 2	0	0
2	A	1	Total C O<br>3 1 2	0	0
2	B	1	Total C O<br>3 1 2	0	0
2	B	1	Total C O<br>3 1 2	0	0
2	B	1	Total C O<br>3 1 2	0	0
2	B	1	Total C O<br>3 1 2	0	0
2	B	1	Total C O<br>3 1 2	0	0

- Molecule 3 is CHLORIDE ION (three-letter code: CL) (formula: Cl).

Mol	Chain	Residues	Atoms	ZeroOcc	AltConf
3	B	2	Total Cl<br>2 2	0	0
3	A	2	Total Cl<br>2 2	0	0

- Molecule 4 is water.

Mol	Chain	Residues	Atoms	ZeroOcc	AltConf
4	A	311	Total O<br>311 311	0	0
4	B	298	Total O<br>298 298	0	0

CONFIDENTIAL VALIDATION REPORT

3 Residue-property plots (i)

These plots are drawn for all protein, RNA and DNA chains in the entry. The first graphic for a chain summarises the proportions of errors displayed in the second graphic. The second graphic shows the sequence view annotated by issues in geometry and electron density. Residues are color-coded according to the number of geometric quality criteria for which they contain at least one outlier: green = 0, yellow = 1, orange = 2 and red = 3 or more. A red dot above a residue indicates a poor fit to the electron density (RSRZ > 2). Stretches of 2 or more consecutive residues without any outlier are shown as a green connector. Residues present in the sample, but not in the model, are shown in grey.

• Molecule 1: Botulinum neurotoxin FA binding domain

Chain A:

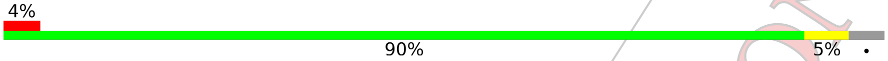

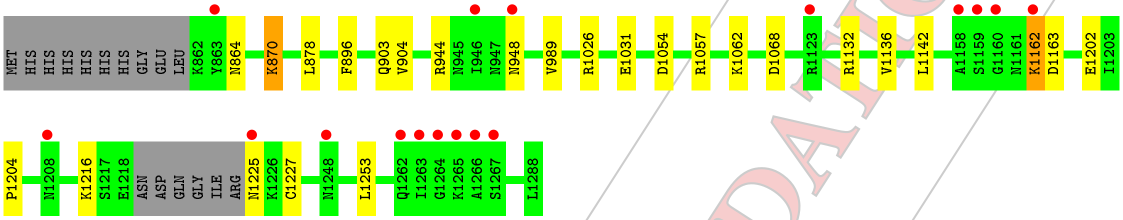

• Molecule 1: Botulinum neurotoxin FA binding domain

Chain B:

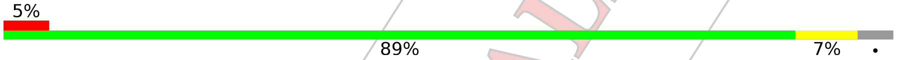

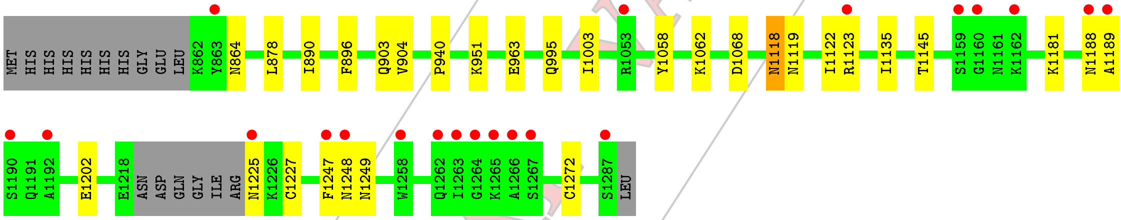

CONFIDENTIAL VALIDATION REPORT

4 Data and refinement statistics ⓘ

Property	Value	Source
Space group	P 4 2 2	Depositor
Cell constants<br>a, b, c, α, β, γ	118.01Å 118.01Å 173.83Å<br>90.00° 90.00° 90.00°	Depositor
Resolution (Å)	118.01 – 1.95<br>118.01 – 1.95	Depositor<br>EDS
% Data completeness<br>(in resolution range)	100.0 (118.01-1.95)<br>100.0 (118.01-1.95)	Depositor<br>EDS
R_{merge}	(Not available)	Depositor
R_{sym}	(Not available)	Depositor
$< I/\sigma(I) >$ [1]	2.29 (at 1.95Å)	Xtriage
Refinement program	PHENIX (1.10.1_2155: ???)	Depositor
R, R_{free}	0.179 , 0.212<br>0.178 , 0.211	Depositor<br>DCC
R_{free} test set	4501 reflections (5.01%)	DCC
Wilson B-factor (Å^2)	25.8	Xtriage
Anisotropy	0.282	Xtriage
Bulk solvent k_{sol}(e/Å^3), B_{sol}(Å^2)	0.37 , 48.5	EDS
L-test for twinning[2]	$< \|L\| >$ = 0.50, $< L^2 >$ = 0.33	Xtriage
Estimated twinning fraction	No twinning to report.	Xtriage
F_o,F_c correlation	0.96	EDS
Total number of atoms	7553	wwPDB-VP
Average B, all atoms (Å^2)	29.0	wwPDB-VP

Xtriage's analysis on translational NCS is as follows: *The largest off-origin peak in the Patterson function is 2.86% of the height of the origin peak. No significant pseudotranslation is detected.*

[1] Intensities estimated from amplitudes.

[2] Theoretical values of $< |L| >$, $< L^2 >$ for acentric reflections are 0.5, 0.333 respectively for untwinned datasets, and 0.375, 0.2 for perfectly twinned datasets.

CONFIDENTIAL VALIDATION REPORT

5 Model quality (i)

5.1 Standard geometry (i)

Bond lengths and bond angles in the following residue types are not validated in this section: FMT, CL

The Z score for a bond length (or angle) is the number of standard deviations the observed value is removed from the expected value. A bond length (or angle) with $|Z| > 5$ is considered an outlier worth inspection. RMSZ is the root-mean-square of all Z scores of the bond lengths (or angles).

Mol	Chain	Bond lengths		Bond angles	
		RMSZ	#|Z| >5	RMSZ	#|Z| >5
1	A	0.37	0/3534	0.59	1/4770 (0.0%)
1	B	0.37	0/3525	0.57	0/4759
All	All	0.37	0/7059	0.58	1/9529 (0.0%)

There are no bond length outliers.

All (1) bond angle outliers are listed below:

Mol	Chain	Res	Type	Atoms	Z	Observed(°)	Ideal(°)
1	A	1253	LEU	CA-CB-CG	5.64	128.27	115.30

There are no chirality outliers.

There are no planarity outliers.

5.2 Too-close contacts (i)

In the following table, the Non-H and H(model) columns list the number of non-hydrogen atoms and hydrogen atoms in the chain respectively. The H(added) column lists the number of hydrogen atoms added and optimized by MolProbity. The Clashes column lists the number of clashes within the asymmetric unit, whereas Symm-Clashes lists symmetry related clashes.

Mol	Chain	Non-H	H(model)	H(added)	Clashes	Symm-Clashes
1	A	3458	0	3411	16	0
1	B	3449	0	3393	16	0
2	A	18	0	6	2	0
2	B	15	0	5	1	0
3	A	2	0	0	0	0
3	B	2	0	0	0	0
4	A	311	0	0	6	0
4	B	298	0	0	1	0

Continued on next page...

CONFIDENTIAL VALIDATION REPORT

Continued from previous page...

Mol	Chain	Non-H	H(model)	H(added)	Clashes	Symm-Clashes
All	All	7553	0	6815	34	0

The all-atom clashscore is defined as the number of clashes found per 1000 atoms (including hydrogen atoms). The all-atom clashscore for this structure is 2.

All (34) close contacts within the same asymmetric unit are listed below, sorted by their clash magnitude.

Atom-1	Atom-2	Interatomic distance (Å)	Clash overlap (Å)
1:A:864:ASN:HB3	1:A:1068:ASP:OD1	1.91	0.70
1:B:864:ASN:HB3	1:B:1068:ASP:OD1	1.91	0.69
1:A:903:GLN:NE2	4:A:1403:HOH:O	2.28	0.65
1:B:1247:PHE:O	1:B:1249:ASN:N	2.34	0.61
1:B:1122:ILE:HG23	1:B:1123:ARG:HD2	1.84	0.59
1:B:1181:LYS:NZ	1:B:1202:GLU:OE1	2.36	0.58
2:B:1303:FMT:H	4:B:1489:HOH:O	2.04	0.57
1:B:878:LEU:HD11	1:B:904:VAL:HG11	1.86	0.56
1:A:878:LEU:HD11	1:A:904:VAL:HG11	1.88	0.55
1:A:1026:ARG:NH2	4:A:1408:HOH:O	2.39	0.55
1:A:1054:ASP:HB3	1:A:1057:ARG:HG2	1.88	0.54
1:A:1216:LYS:NZ	4:A:1411:HOH:O	2.43	0.52
1:A:1202:GLU:HG3	1:A:1204:PRO:HD2	1.90	0.52
2:A:1302:FMT:O2	2:A:1303:FMT:O2	2.28	0.51
1:A:944:ARG:HD3	1:B:1145:THR:HG21	1.94	0.49
1:A:989:VAL:HG22	1:A:1031:GLU:HB3	1.96	0.48
1:B:878:LEU:HB2	1:B:890:ILE:HD11	1.95	0.48
1:B:1118:ASN:OD1	1:B:1119:ASN:N	2.47	0.47
1:A:944:ARG:NE	4:A:1416:HOH:O	2.48	0.46
1:B:1227:CYS:HA	1:B:1272:CYS:O	2.16	0.46
1:B:903:GLN:HG2	1:B:1062:LYS:HD3	1.98	0.46
1:A:870:LYS:HE3	4:A:1419:HOH:O	2.17	0.45
1:B:995:GLN:HA	1:B:1003:ILE:HD11	1.99	0.44
1:B:951:LYS:HD3	1:B:963:GLU:OE2	2.17	0.43
1:A:1136:VAL:HG22	1:A:1142:LEU:HG	2.00	0.43
1:B:1135:ILE:HG23	1:B:1145:THR:HG22	2.00	0.42
1:A:1225:ASN:ND2	1:A:1227:CYS:H	2.18	0.42
1:A:1162:LYS:HD2	1:A:1163:ASP:N	2.35	0.42
1:B:940:PRO:HG3	1:B:1058:TYR:CE1	2.55	0.42
1:B:896:PHE:CD2	1:B:904:VAL:HG12	2.55	0.41
1:A:903:GLN:HG2	1:A:1062:LYS:HD3	2.03	0.41
1:B:1188:ASN:O	1:B:1189:ALA:HB3	2.21	0.40
2:A:1302:FMT:H	4:A:1454:HOH:O	2.20	0.40

Continued on next page...

CONFIDENTIAL VALIDATION REPORT

Continued from previous page...

Atom-1	Atom-2	Interatomic distance (Å)	Clash overlap (Å)
1:A:896:PHE:CD2	1:A:904:VAL:HG12	2.56	0.40

There are no symmetry-related clashes.

5.3 Torsion angles (i)

5.3.1 Protein backbone (i)

In the following table, the Percentiles column shows the percent Ramachandran outliers of the chain as a percentile score with respect to all X-ray entries followed by that with respect to entries of similar resolution.

The Analysed column shows the number of residues for which the backbone conformation was analysed, and the total number of residues.

Mol	Chain	Analysed	Favoured	Allowed	Outliers	Percentiles	
1	A	419/437 (96%)	406 (97%)	12 (3%)	1 (0%)	52	43
1	B	418/437 (96%)	406 (97%)	11 (3%)	1 (0%)	52	43
All	All	837/874 (96%)	812 (97%)	23 (3%)	2 (0%)	52	43

All (2) Ramachandran outliers are listed below:

Mol	Chain	Res	Type
1	A	948	ASN
1	B	1248	ASN

5.3.2 Protein sidechains (i)

In the following table, the Percentiles column shows the percent sidechain outliers of the chain as a percentile score with respect to all X-ray entries followed by that with respect to entries of similar resolution.

The Analysed column shows the number of residues for which the sidechain conformation was analysed, and the total number of residues.

Mol	Chain	Analysed	Rotameric	Outliers	Percentiles	
1	A	387/400 (97%)	384 (99%)	3 (1%)	86	85
1	B	386/400 (96%)	384 (100%)	2 (0%)	92	91
All	All	773/800 (97%)	768 (99%)	5 (1%)	88	89

CONFIDENTIAL VALIDATION REPORT

All (5) residues with a non-rotameric sidechain are listed below:

Mol	Chain	Res	Type
1	A	870	LYS
1	A	1132	ARG
1	A	1162	LYS
1	B	1118	ASN
1	B	1225	ASN

Some sidechains can be flipped to improve hydrogen bonding and reduce clashes. All (2) such sidechains are listed below:

Mol	Chain	Res	Type
1	A	920	ASN
1	B	1246	GLN

5.3.3 RNA (i)

There are no RNA molecules in this entry.

5.4 Non-standard residues in protein, DNA, RNA chains (i)

There are no non-standard protein/DNA/RNA residues in this entry.

5.5 Carbohydrates (i)

There are no carbohydrates in this entry.

5.6 Ligand geometry (i)

Of 15 ligands modelled in this entry, 4 are monoatomic - leaving 11 for Mogul analysis.

In the following table, the Counts columns list the number of bonds (or angles) for which Mogul statistics could be retrieved, the number of bonds (or angles) that are observed in the model and the number of bonds (or angles) that are defined in the chemical component dictionary. The Link column lists molecule types, if any, to which the group is linked. The Z score for a bond length (or angle) is the number of standard deviations the observed value is removed from the expected value. A bond length (or angle) with $|Z| > 2$ is considered an outlier worth inspection. RMSZ is the root-mean-square of all Z scores of the bond lengths (or angles).

Mol	Type	Chain	Res	Link	Bond lengths			Bond angles		
					Counts	RMSZ	$\#\|Z\|>2$	Counts	RMSZ	$\#\|Z\|>2$
2	FMT	A	1301	-	0,2,2	0.00	-	0,1,1	0.00	-

CONFIDENTIAL VALIDATION REPORT

Mol	Type	Chain	Res	Link	Bond lengths			Bond angles		
					Counts	RMSZ	#|Z| > 2	Counts	RMSZ	#|Z| > 2
2	FMT	A	1302	-	0,2,2	0.00	-	0,1,1	0.00	-
2	FMT	A	1303	-	0,2,2	0.00	-	0,1,1	0.00	-
2	FMT	A	1304	-	0,2,2	0.00	-	0,1,1	0.00	-
2	FMT	A	1305	-	0,2,2	0.00	-	0,1,1	0.00	-
2	FMT	A	1306	-	0,2,2	0.00	-	0,1,1	0.00	-
2	FMT	B	1301	-	0,2,2	0.00	-	0,1,1	0.00	-
2	FMT	B	1302	-	0,2,2	0.00	-	0,1,1	0.00	-
2	FMT	B	1303	-	0,2,2	0.00	-	0,1,1	0.00	-
2	FMT	B	1304	-	0,2,2	0.00	-	0,1,1	0.00	-
2	FMT	B	1305	-	0,2,2	0.00	-	0,1,1	0.00	-

In the following table, the Chirals column lists the number of chiral outliers, the number of chiral centers analysed, the number of these observed in the model and the number defined in the chemical component dictionary. Similar counts are reported in the Torsion and Rings columns. '-' means no outliers of that kind were identified.

Mol	Type	Chain	Res	Link	Chirals	Torsions	Rings
2	FMT	A	1301	-	-	0/0/0/0	0/0/0/0
2	FMT	A	1302	-	-	0/0/0/0	0/0/0/0
2	FMT	A	1303	-	-	0/0/0/0	0/0/0/0
2	FMT	A	1304	-	-	0/0/0/0	0/0/0/0
2	FMT	A	1305	-	-	0/0/0/0	0/0/0/0
2	FMT	A	1306	-	-	0/0/0/0	0/0/0/0
2	FMT	B	1301	-	-	0/0/0/0	0/0/0/0
2	FMT	B	1302	-	-	0/0/0/0	0/0/0/0
2	FMT	B	1303	-	-	0/0/0/0	0/0/0/0
2	FMT	B	1304	-	-	0/0/0/0	0/0/0/0
2	FMT	B	1305	-	-	0/0/0/0	0/0/0/0

There are no bond length outliers.

There are no bond angle outliers.

There are no chirality outliers.

There are no torsion outliers.

There are no ring outliers.

3 monomers are involved in 3 short contacts:

Mol	Chain	Res	Type	Clashes	Symm-Clashes
2	A	1302	FMT	2	0
2	A	1303	FMT	1	0
2	B	1303	FMT	1	0

CONFIDENTIAL VALIDATION REPORT

5.7 Other polymers (i)

There are no such residues in this entry.

5.8 Polymer linkage issues (i)

There are no chain breaks in this entry.

CONFIDENTIAL VALIDATION REPORT

6 Fit of model and data (i)

6.1 Protein, DNA and RNA chains (i)

In the following table, the column labelled '#RSRZ> 2' contains the number (and percentage) of RSRZ outliers, followed by percent RSRZ outliers for the chain as percentile scores relative to all X-ray entries and entries of similar resolution. The OWAB column contains the minimum, median, 95th percentile and maximum values of the occupancy-weighted average B-factor per residue. The column labelled 'Q< 0.9' lists the number of (and percentage) of residues with an average occupancy less than 0.9.

Mol	Chain	Analysed	<RSRZ>	#RSRZ>2			OWAB(Å^2)	Q<0.9
1	A	421/437 (96%)	0.27	17 (4%)	42	53	15, 26, 50, 79	0
1	B	420/437 (96%)	0.36	21 (5%)	32	44	15, 27, 58, 89	0
All	All	841/874 (96%)	0.31	38 (4%)	37	48	15, 26, 52, 89	0

All (38) RSRZ outliers are listed below:

Mol	Chain	Res	Type	RSRZ
1	B	1263	ILE	7.2
1	B	1266	ALA	7.1
1	B	1267	SER	5.9
1	A	1159	SER	5.8
1	A	1265	LYS	5.7
1	A	863	TYR	5.3
1	A	1266	ALA	5.3
1	A	1160	GLY	5.2
1	B	1248	ASN	5.2
1	B	1265	LYS	5.1
1	A	1158	ALA	4.7
1	B	1160	GLY	4.5
1	A	1263	ILE	4.2
1	A	1264	GLY	4.0
1	B	1264	GLY	3.8
1	B	1188	ASN	3.7
1	B	1162	LYS	3.5
1	B	1189	ALA	3.3
1	B	1190	SER	3.1
1	B	863	TYR	3.1
1	B	1258	TRP	3.0
1	A	948	ASN	2.9
1	B	1225	ASN	2.7
1	A	1248	ASN	2.7

Continued on next page...

CONFIDENTIAL VALIDATION REPORT

Continued from previous page...

Mol	Chain	Res	Type	RSRZ
1	B	1159	SER	2.6
1	A	1225	ASN	2.5
1	B	1287	SER	2.4
1	B	1262	GLN	2.4
1	A	1123	ARG	2.4
1	B	1053	ARG	2.4
1	A	946	ILE	2.4
1	A	1162	LYS	2.2
1	B	1192	ALA	2.2
1	A	1267	SER	2.2
1	A	1208	ASN	2.2
1	B	1247	PHE	2.2
1	B	1123	ARG	2.2
1	A	1262	GLN	2.2

6.2 Non-standard residues in protein, DNA, RNA chains ⓘ

There are no non-standard protein/DNA/RNA residues in this entry.

6.3 Carbohydrates ⓘ

There are no carbohydrates in this entry.

6.4 Ligands ⓘ

In the following table, the Atoms column lists the number of modelled atoms in the group and the number defined in the chemical component dictionary. LLDF column lists the quality of electron density of the group with respect to its neighbouring residues in protein, DNA or RNA chains. The B-factors column lists the minimum, median, 95th percentile and maximum values of B factors of atoms in the group. The column labelled 'Q< 0.9' lists the number of atoms with occupancy less than 0.9.

Mol	Type	Chain	Res	Atoms	RSCC	RSR	LLDF	B-factors(Å^2)	Q<0.9
2	FMT	B	1302	3/3	0.83	0.37	22.19	35,35,36,38	0
2	FMT	B	1305	3/3	0.98	0.32	20.65	27,27,28,38	0
2	FMT	A	1305	3/3	0.96	0.39	16.61	32,32,37,37	0
2	FMT	A	1304	3/3	0.94	0.35	14.02	27,27,34,35	0
2	FMT	A	1303	3/3	0.88	0.26	11.83	27,27,30,38	0
2	FMT	A	1302	3/3	0.97	0.16	2.34	28,28,29,37	0
2	FMT	B	1303	3/3	0.92	0.15	2.07	25,25,31,32	0

Continued on next page...

CONFIDENTIAL VALIDATION REPORT

Continued from previous page...

Mol	Type	Chain	Res	Atoms	RSCC	RSR	LLDF	B-factors(Å^2)	Q<0.9
2	FMT	B	1301	3/3	0.98	0.13	2.04	30,30,33,33	0
2	FMT	B	1304	3/3	0.75	0.18	1.09	34,34,40,46	0
3	CL	B	1307	1/1	0.98	0.12	-0.83	30,30,30,30	0
3	CL	A	1308	1/1	1.00	0.08	-1.50	32,32,32,32	0
3	CL	B	1306	1/1	0.98	0.06	-	34,34,34,34	0
3	CL	A	1307	1/1	0.98	0.07	-	34,34,34,34	0
2	FMT	A	1306	3/3	0.82	0.38	-	31,31,40,41	0
2	FMT	A	1301	3/3	0.42	0.41	-	44,44,45,46	0

6.5 Other polymers (i)

There are no such residues in this entry.

CONFIDENTIAL VALIDATION REPORT
